# Exploring MQTT Broker-Based, End-to-End Models for Security and Efficiency

**DOI:** 10.3390/s25175308

**Published:** 2025-08-26

**Authors:** Hung-Yu Chien, An-Tong Shih, Yuh-Ming Huang

**Affiliations:** 1 Department of Information Management, National Chinan University, Nantou County 54561, Taiwan; 2 Department of Computer Science & Information Engineering, National Chinan University, Nantou County 54561, Taiwan; s112321902@mail1.ncnu.edu.tw (A.-T.S.); ymhuang@csie.ncnu.edu.tw (Y.-M.H.)

**Keywords:** MQTT, Mosquitto, ECDH, enhanced authentication, end to end

## Abstract

**Highlights:**

**What are the main findings?**

- Highlight the double-encryption issue of the conventional MQTT broker-based, end-to-end (E2E) models.
- Highlight the heavy broker-decrypt-re-encrypt overhead undermining the MQTT efficiency strengths.
- Explore new MQTT broker-based E2E models, which aim to preserve high efficiency and security goals.

**What is the implication of the main finding?**

- Both the group key-based approach and the client–broker channel, Integrity-only approach are promising solutions for securing MQTT systems while preserving the efficiency strength.
- The proposed approaches are fully compatible with MQTT 5.0 and have been implemented using MQTT 5.0 APIs.

**Abstract:**

MQTT is a publisher–broker–subscriber architecture in which a broker forwards the messages to interested subscribers, which facilitates the broker’s capacity to peek at the message contents; therefore, both academia and industry design and develop end-to-end (E2E) channels to protect the privacy against a curious broker which honestly follows the protocols but would peek at the contents for its benefits. However, we notice that the double-encryption issue of the conventional MQTT broker-based E2E models and the heavy broker-decrypt-re-encrypt overhead undermine MQTT efficiency strengths. In this study, we highlight the weaknesses, propose several solutions, implement the schemes, and experiment with them in the simulated scenarios. Security analysis and formal security proofs are verified to ensure the security goals. The analysis and the evaluations on the implementations confirm both the group key-based approach and the client–broker-channel, integrity-only approach could improve the efficiency performance while preserving security strengths.

## 1. Introduction

MQTT (message queuing telemetry transport) [1,2], as one of the most popular Internet of Things (IoT) communication protocols, has been widely deployed around the globe. However, its publisher–broker–subscriber architecture allows the broker to peek at the message content, even if a TLS authentication is activated between clients and a broker.

**Figure 1:**
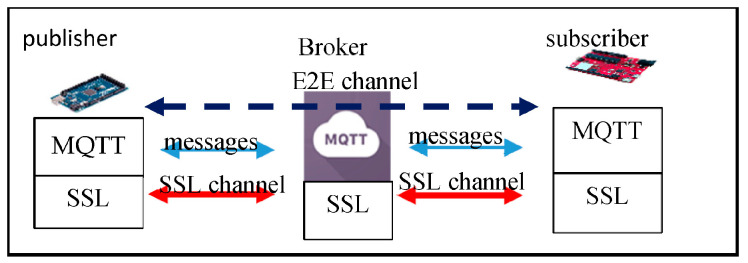
The MQTT-SSL model.

To protect the message privacy from curious brokers, several works like [3,4,5,6,7,8] propose their solutions for the end-to-end (E2E) channel building between a publisher and a subscriber; in such settings, a publisher and a subscriber would build an E2E channel, in addition to the client–broker secure channel. Figure 1 shows the MQTT E2E architecture. Conventional MQTT standards (MQTT 5.0 [2], MQTT 3.1 [1] and its previous versions) encourage users to activate SSL/TLS on the brokers or design their customized secure channel between clients and a broker to protect the security between clients and the broker.

However, we find that this MQTT E2E model would result in a double-encryption-overhead issue. In Figure 1, assume publisher $P_{A}$ publishes a message $M_{A}$ to a group of *n* subscribers (say $S_{i}$, for i=1~n); $P_{A}$ first encrypts $M_{A}$ using the E2E key between $P_{A}$ and $S_{i}$ for i=1~n) to get ${E(M_{A})}_{{E2E}_{P_{A},S_{i}}}$, and then re-encrypts each encryption again using the SSL key between $P_{A}$ and B to get $M^{\prime \prime }={E({E(M_{A})}_{{E2E}_{P_{A},S_{i}}})}_{{SSL}_{P_{A},B}}$; upon receiving the double-encrypted message $M^{\prime \prime }$, the broker first decrypts it to get ${E(M_{A})}_{{E2E}_{P_{A},S_{i}}}$, and then re-encrypts it using the SSL key between it and $S_{i}$ to get $M^{\prime \prime \prime }={E({E(M_{A})}_{{E2E}_{P_{A},S_{i}}})}_{{SSL}_{B,S_{i}}}$; finally, $S_{i}$ decrypts $M^{\prime \prime \prime }$ twice to get the plaintext.

In this conventional MQTT E2E model, we would like to point out two critical weaknesses; one is the double-encryption issue from $P_{A}$ and *B*, and from *B* to $S_{i}$; the other is the broker-decrypt-re-encrypt overhead, of which the broker *B*, for each MQTT message, decrypts once and re-encrypts again *n* times, which is a huge overhead. The two critical weaknesses undermine the efficiency strengths of conventional MQTT E2E systems.

In this study, we point out the weaknesses, propose our solutions, evaluate the performance, and prove the security. The rest of this work is organized as follows. Section 2 discusses the related work. Section 3 introduces our efficiency-enhanced MQTT E2E solutions. Section 4 presents our implementation and evaluates the performance. Section 5 analyzes the security and gives a formal security proof. Section 6 states our conclusions and future work.

## 2. Related Work

Most of the existing MQTT security publications, like [4,5,7,8], tackle the security of MQTT 3.1 and its earlier versions, and only a few, like [3,6], focus on MQTT 5.0. MQTT 5.0 was released in 2019; its main enhancements include enhanced authentication, user property, client–server interaction, user data, etc., to provide more flexibility in designing customized functions and security supports; therefore, it is much more convenient to design and implement customized functions like authentication, key agreement, publisher–subscriber interaction, etc.

Due to MQTT’s publisher–broker–subscriber architecture, a broker acts as a message forwarder between a publisher and its subscribers, which facilitates the broker’s capacity to peek at the message contents. This would be a serious privacy threat, especially when the clients and the broker do not belong to the same authority. To address this weakness, some works [3,4,5,6,7,8] focus on building a secure E2E channel between publishers and subscribers: the messages from the publisher and its subscribers are encrypted using the E2E channel keys.

Chien [3] proposes designing two client-initiated E2E scenarios: one is for the publisher-initiated E2E scenario and the other is the subscriber-initiated E2E scenario. Mektoubi et al.’s E2E solution [7] adopts the topic certificate, of which the corresponding public key is used to encrypt the messages for the topic while the private key is used to decrypt the encryptions. This approach demands a huge overhead from the clients, and the private key distribution raises a challenge.

In the MQTLS scheme [4], a publisher initiates its E2E key distribution using the publisher’s–subscriber’s ephemeral Diffie–Hellman session keys, and OpenSSL-based extensions are used to facilitate the E2E topic key distribution. However, their extension implementations do not consider MQTT 5.0 compatibility. Efficiency improvement using a broker-based group key is not applied. Neither the double-encryption issue nor the decrypt-re-encrypt overhead is considered. Their E2E channel establishment is not publisher–subscriber decoupling, either.

SEEMQTT’s E2E scheme [5], based on the secret sharing mechanism, allows a publisher to delegate its decryption authority to a pool of key stores. Those designated subscribers contact the pool of key stores, get verification, and then recover the decryption key. This solution lets the publisher delegate its authority to verify the subscribers to a pool of key stores, which results in lots of extra overhead.

Chein et al. [6] design, implement, and evaluate the performance of their MQTT E2E scheme for MQTT 5.0; The evaluation confirms that building an E2E channel between a publisher and a subscriber only adds non-significant connection latency.

Wang and Chien [8], based on the MQTT E2E channel, improve the security and the efficiency of the MQTT over the air (OTA) updating mechanism. However, the double-encryption issue is not addressed.

In addition to the above schemes, there are some schemes related to our broker-based E2E schemes. Eugster et al. [9] focus on discussing the decoupling properties in terms of space, time, and synchronization for those publish/subscribe interaction schemes. Regarding the decoupling feature of MQTT, we would like to categorize it into two situations. One is the publisher–subscriber E2E-connection decoupling which means that, during the publisher–subscriber E2E channel building process, the two entities need not interact with each other. The other one is message-forwarding decoupling, which means that, when a publisher/subscriber publishes/receives a message, they do not need to be aware or interact with each other. In these senses, the models we discuss and propose in this study satisfy the message-forwarding decoupling but not the publisher–subscriber E2E-connection decoupling.

Now we briefly introduce the potentials/supports of facilitating some coupling designs in MQTT 5.0. MQTT 5.0 supports the enhanced authentication framework (EAF) and User properties, where user properties are user-defined meta shared among publishers, brokers, and subscribers. EAF contains CONNECT, DISCONNECT, and AUTH API; in these APIs, the new fields auth_id, auth_data, and User properties facilitate the customized security designs and the conventional client–server interactions between a publisher and a subscriber in the connection phase. By subscribing to the designated topics and properly setting the fields—the response topic and the correlation data—a publisher and a subscriber can perform conventional client–server interaction between them. The above introduction implies that MQTT 5.0 facilitates the publisher–subscriber E2E-connection coupling and publisher–subscriber message coupling. Interested readers are referred to [2,10,11,12,13] for detailed introductions and to [6] for the implementation examples. That is, MQTT 5.0 facilitates the support of both decoupling designs and coupling designs; the choice depends on the application requirements.

Buccafurri et al. [14] systematically examine the applicability of existent mechanisms for ensuring the MQTT E2E flow integrity; they discuss the MQTT E2E flow integrity in terms of formally-defined properties like Completeness, Correctness, and Liveness. Their MQTT-I scheme combines the message-digest hashing, multiple-topic Merkle hash tree, and round-based signature/verification to amortize the extra signature/verification cost. However, we notice two critical differences between MQTT-I E2E solutions and the MQTT broker-based E2E schemes we focus on here. First, we are concerned with E2E privacy and data integrity protection against a curious broker, but MQTT-I concerns the flow integrity (the integrity property is different from others), but not the privacy. Many works like [3,4,5,6,7,8] have highlighted the importance of privacy protection against a curious broker. Second, in their simulations and evaluation [14], they do not consider the performance impact of increasing the number of clients on the performance of publisher/broker/subscriber; however, many existing studies have confirmed that the increasing number of clients really has great impact on the overall performance, especially when the customized broker has some extra overheads.

MQTT-SN (MQTT for sensor networks) [15] is a more lightweight variant of MQTT; it adjusts MQTT’s features to fit those scenarios and environments where the communications are more restricted. Park and Nam [16] concern the E2E security (privacy and integrity) against a curious broker for MQTT-SN; they employ entity certificates, topic certificates, and public/private keys to facilitate the establishment of the session keys between entities. Their ideas are applicable to the MQTT systems, but they do not concern the double encryption issue and the decrypt then re-encrypt issue.

Singh et al. [17] augment existing MQTT protocols with key/ciphertext policy-attribute-based encryption (KP/CP-ABE) [18,19] for securing MQTT applications in sensor network environments. This attribute-based, encryption-based (ABE-based) approach can be applied and extended to secure both client–broker channels and publisher–subscriber channels; however, this approach has the key weaknesses of poor performance and scalability, especially when the number of attributes increases. This weakness should be seriously tackled as MQTT is targeted for IoT systems where the number of clients and the number of topics are scalable.

In a short summary, even though there are several MQTT E2E schemes proposed, implemented, and evaluated, we notice that they do not focus on the double-encryption issue and the impact of the broker-decrypt-re-encrypt overhead, and do not systematically explore potential approaches to tackle these challenges while preserving the desirable security properties. Therefore, we will discuss and tackle these challenges.

While MQTT remains one of the most widely adopted messaging protocols in IoT due to its lightweight publish/subscribe model and TCP-based reliability, several alternative protocols have been developed to address specific deployment scenarios. AMQP (advanced message queuing protocol) [20] offers robust messaging features such as queuing, routing, and transaction support, making it more suitable for enterprise environments rather than resource-constrained devices. Meanwhile, CoAP over DTLS [21] provides a RESTful communication model optimized for constrained nodes and networks, using UDP and DTLS to achieve lightweight, secure, request/response messaging. The data distribution service (DDS) [22] for real-time systems is an object management group (OMG) machine-to-machine middleware standard that aims to enable dependable, high-performance, interoperable, real-time, scalable data exchanges using a publish–subscribe pattern.

Some other studies are related in a broader sense. Ciphertext matching like [23] could be applied to a general sub/subsystem to ensure the event privacy and the subscription privacy against a curious broker; even though MQTT E2E study also concern the privacy of message contents against a curious broker, both the subscribed topics and the topic of a published message are explicitly sent to the broker in MQTT systems.

Brokerless extensions eliminate the central broker by enabling direct peer-to-peer message routing. One approach is multicast-based MQTT over software-defined networks like Park et al.’s DM-MQTT [24]; DM-MQTT reduces data transfer delays by establishing bidirectional SDN (software defined networking) multicast trees between the publishers and the subscribers by bypassing the centralized broker. Shahriet et al. [25], based on SDN, further apply MQTT 5.0’s User properties to specify the real-time requirements to instantiate corresponding network reservations to achieve the desirable real-time transmissions. ZeroMQ [26] is a lightweight, brokerless messaging library offering direct pub/sub, request/reply, and pipeline patterns with low latency. Our approach aims at improving efficiency by adjusting the MQTT broker-based E2E models; while the brokerless approach adopts transmission reservation facilities (like SDN) to improve the transmission latency. These two approaches are complementary to each other; the idea from either approach can be combined with the other to improve efficiency further.

NIST SP 800-207 “Zero Trust Architecture (ZTA)” [27] enforces a “never trust, always verify” approach to minimize the risk of unauthorized access and lateral movement within networks by continuously validating identities, devices, and contextual signals before granting access to resources. The key functions/components include policy decision point (PDP), policy enforcement point (PEP), resource access policies, and identity management. It provides a complete guide for security system designs and implementations.

The above works are all related to IoT protocols, and this paper focuses on MQTT broker-based E2E systems’ security and efficiency. Therefore, we will only discuss MQTT and MQTT-SN in the rest of this paper. We systematically examine the efficiency of the conventional MQTT broker-based E2E model’s security properties and efficiencies, and explore potential approaches to enhance the efficiencies while preserving the desirable security properties.

There exist some popular mechanisms which formally analyze the security properties of security protocols. The first category of these mechanisms is based on some mathematical reduction proof in the specified formal models. The second category includes those mechanisms [28,29,30,31,32,33,34,35,36] that provide software tools that formally execute logic reasoning on formal specifications of security protocols. Both Burrows–Abadi–Needham (BAN) logic [28] and the AVISPA [29,30] are two popular formal tools of the second category. The BAN logic is a set of rules for defining and analyzing information exchange protocols, and has been successfully applied to protocol conformance testing; however, there are many reports like [32,33] on the limitations, weaknesses, and failures of the BAN logic. On the contrary, the AVISPA is designed to simulate and verify cryptographic protocols formally; it provides the SPAN protocol simulation [34] to examine the interaction flows formally; it contains various verification tools (like OFMC [35], ATSE) to validate the security goals formally. The high-level protocol specification language (HLPSL) [36] is the formal language used to formally define the protocol role, environment, and security goals in AVISPA. Even though the AVISPA and its tools still have some limitations (for example, the limited support of some algebra calculations), they provide formal specifications of the protocols, of the simulated environments, and of the security goals, which can better capture the security semantics. We, therefore, adopt the AVISPA to verify our schemes.

## 3. Efficiency-Enhanced MQTT E2E Architecture (Model)

Before proposing our efficiency-enhanced MQTT E2E architecture (E-MQTT-E2EA), we review the key features of the conventional MQTT broker-based E2E mechanisms, and then introduce the performance metrics for comparisons.

### 3.1. Threat Model and Assumptions

Here, we define the threat model and the assumptions for the rest of this paper. In our model, the broker is honest but curious, and the clients might be compromised. An honest but curious broker would honestly execute the protocol, but would peek at the content for their own benefit. A compromised client could impersonate other entities, and replay/modify/intercept/forge the messages.

We assume the system has implemented some kind of intrusion detection system to detect and identify a compromised client. We also assume that some proper policy enforcement functions and access controls have been implemented on the broker such that granting the publish/subscribe/connect requests would follow the policies defined for that client.

### 3.2. Performance Challenges of Conventional MQTT Broker-Based E2E Mechanisms

Here, we define the conventional MQTT broker-based E2E schemes, be those MQTT secure-channel building schemes which simultaneously achieve the two functions: (1) the client–broker channel provides mutual authentication and privacy protection (for example, TLS aims at providing secure client–broker channels); (2) a publisher builds E2E channels with its subscribers, and the channel provides mutual authentication and privacy protection. Here, the E2E channel building is coupled in the sense that the publisher and the subscriber interact with each other via a broker. Regarding these conventional MQTT broker-based E2E schemes, they inherit the following issues. To simplify the notations, we do not differentiate the terms MQTT broker-based E2E and MQTT E2E for the rest of this paper, when the semantics are clear.

1. The double-encryption issue. When a publisher publishes a message, it encrypts the message first using the E2E channel key and then re-encrypts the ciphertext again using the publisher–broker channel key. The same issue happens at the broker-subscriber channel.
2. The broker-decrypt-re-encrypt overhead. In the conventional MQTT architectures, a broker needs to decrypt a ciphertext from a publisher using the publisher–broker-channel key, and then re-encrypts the plaintext using its broker–subscriber channel key. If there are many subscribers for the message, it demands lots of re-encryption overhead. This overhead might seem unavoidable as the broker needs to protect the privacy on the broker–subscriber channel. But, it could be improved as the group of subscribers corresponding to the same topic could use the same group key, and, if the MQTT payload in the E2E channel has been encrypted, the re-encryption could be eliminated.

Regarding the performance of an MQTT E2E mechanism, we are concerned about both the security support and efficiency.

1. The E2E channel security, which includes the authentication of the participating entities and the privacy/integrity of the message.
2. The privacy, authentication, and integrity of the data between a client and a broker.
3. The publisher–broker channel overhead.
4. The broker–subscriber channel overhead.
5. The broker overhead.

### 3.3. High-Level Designs of MQTT E2E Mechanisms

Before discussing our ideas, we introduce the notation in Table 1.

**Figure 2:**
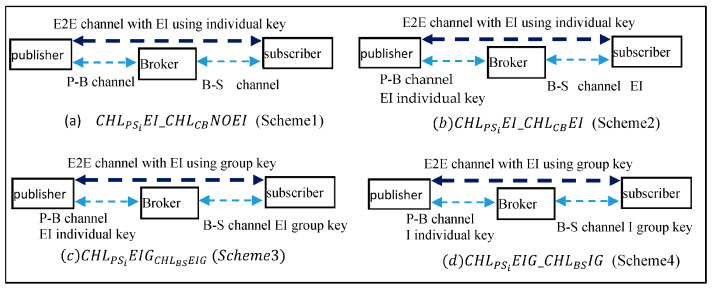
The four MQTT E2E models, where E denotes Encryption, and I denotes Integrity.

Since we are concerned with the performance impact of the MQTT E2E architecture, we explore several ideas for improving the efficiency and evaluate their performance. Figure 2 shows the four channel protection models in the MQTT E2E architecture. All four MQTT E2E models support both authentication and integrity protection on the publisher–subscriber E2E channel, as all MQTT E2E models aim at providing authenticity and integrity for the message transmissions in the E2E channel. But, they differ in the client–broker channel security designs and the broker–subscriber channel key selection.

Because this study focuses on comparing the efficiency improvement by simplifying the client–broker channels and using a group key in some models, we, therefore, simplify the group key being chosen and distributed by the sender (a publisher in the E2E channel for the same topic or the broker in the broker-subscriber channels for the same topic) to simplify the presentation and the prototype implementations.

**Figure 3:**
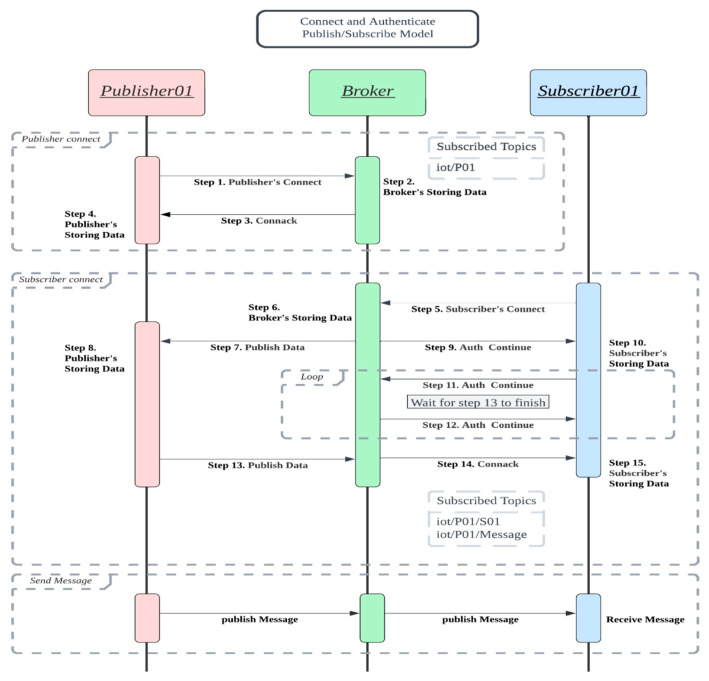
The protocol flows of the four models.

In such a setting, when a publisher P publishes a message for a topic (say ${Topic}_{P})$, P chooses a group key ${GK}_{P}$ and distributes the key during the E2E channel establishment phase. Because our E2E channel establishment phase does not decouple the publisher and the subscriber (it corresponds to the subscriber connect block in Figure 3), the publisher has the opportunity to distribute the encrypted group key (encrypted by the publisher–subscriber Diffie–Hellman session key). Likewise, the broker can choose and distribute the broker–subscriber group key in the subscriber connect phase.

Regarding the dynamic membership of publisher/subscriber and the dynamic connect/disconnect, one solution is described here. The publisher could periodically choose a new group key and distribute it during the subscriber E2E connect phase. This applies to the broker’s choosing and distributing its broker–subscriber group key. When the broker detects and identifies a compromised subscriber (or publisher), it rejects its connection.

Another approach to determine and update the group key is borrowing Park-Nam’s ideas [16]. In Park-Nam’s work [16], two kinds of certificates (entity certificates and topic certificates) and the corresponding private keys are distributed to the corresponding entities. The CDHP-based group keys could be derived using the corresponding public/private keys and some necessary data to differentiate each session. In such a setting, the group key update is by renewing/distributing the corresponding topic certificate and the public/private key to the authorized entities. Interested readers are referred to Park-Nam’s work [16].

Scheme 1-${CHL}_{PS_{i}}EI\_{CHL}_{CB}NOEI$: (1) the E2E channel ${CHL}_{PS_{i}}$ with encryption and integrity check using ${KE2E}_{PS_{i}}$, where the ${KE2E}_{PS_{i}}$ key is set as the individual E2E session key between P and $S_{i}$; (2) the channel ${CHL}_{PB}$ without encryption and integrity; (3) the channel ${CHL}_{BS_{i}}$ without encryption and integrity. This corresponds to the MQTT E2E model without enabling a secure client-broker channel.

Scheme 2-${CHL}_{PS_{i}}EI\_{CHL}_{CB}EI$: (1) the E2E channel ${CHL}_{PS_{i}}$ with encryption and integrity check using ${KE2E}_{PS_{i}}$, where the ${KE2E}_{PS_{i}}$ key is set as the individual E2E session key between P and $S_{i}$; (2) the channel ${CHL}_{PB}$ with encryption and integrity using the key ${SK}_{PB}$, where the key is the individual session key; (3) the channel ${CHL}_{BS_{i}}$ with encryption and integrity using the key ${SK}_{BS_{i}}$, where the key is the individual session key. This corresponds to the conventional MQTT E2E model with a secure client–broker channel (like MQTT E2E with TLS client-broker channel).

Scheme 3-${CHL}_{PS_{i}}EIG\_{CHL}_{BS}EIG$: (1) the E2E channel ${CHL}_{PS_{i}}$ with encryption and integrity check using ${KE2E}_{PS_{i}}={GK}_{P}$, where the ${KE2E}_{PS_{i}}$ key is set as the group key chosen and distributed by P; (2) the channel ${CHL}_{PB}$ with encryption and integrity using the key ${SK}_{PB}$, where the key is the individual session key; (3) the channel ${CHL}_{BS_{i}}$ with encryption and integrity using the key ${SK}_{BS_{i}}={GK}_{B}$, where the key is the group key chosen and distributed by *B*. This new model tries to improve the conventional MQTT E2E model performance using the mechanism: it replaces the individual broker-subscriber session key with the corresponding group key, such that the broker can encrypt once for all the subscribers for the same topic.

Scheme 4-${CHL}_{PS_{i}}EIG\_{CHL}_{BS}IG$: (1) the E2E channel ${CHL}_{PS_{i}}$ with encryption and integrity check using ${KE2E}_{PS_{i}}={GK}_{P}$, where the ${KE2E}_{PS_{i}}$ key is set as the group key chosen and distributed by P; (2) the channel ${CHL}_{PB}$ with the integrity only using the key ${SK}_{PB}$, where the key is the individual session key; (3) the channel ${CHL}_{BS_{i}}$ with the integrity only using the key ${SK}_{BS_{i}}={GK}_{B}$, where the key is the group key chosen and distributed by *B*. This model tries to improve the previous model’s performance further using the two mechanisms: (a) it eliminates the double-encryption issue on the client–broker channels by providing only authentication and integrity checks on the client–broker channel; the rationale for this design is that, as the publisher has encrypted the message using the E2E key, it only needs to ensure the authentication and integrity on the publisher–broker channel; (b) a similar rationale applied on the broker–subscriber channel. Here, we note that all the sensitive data in the E2E channel should be encrypted using the E2E key, and only non-sensitive data fields can be assigned to MQTT plaintext fields. This ensures that the receiver can still authenticate the source and the integrity of the received messages.

The rationale behind the above scheme notations could be explained as follows. ${CHL}_{PS_{i}}EI\_{CHL}_{CB}NOEI$ refers to that (1) the channel ${CHL}_{PS_{i}}$ with encryption and integrity using individual session keys—EI, and (2) the channel ${CHL}_{CB}$ between a Client (*C*) and its Broker (*B*) without (NO) encryption integrity—NOEI. ${CHL}_{PS_{i}}EIG\_{CHL}_{BS}IG$ refers to that (1) the channel ${CHL}_{PS_{i}}$ with encryption and integrity using Group key—EIG, and (2) the channel ${CHL}_{BS}$ between a broker (*B*) with its subscribers (*S*) with integrity using Group key (*G*)—IG. Other notations could be inferred similarly.

Scheme 1 (${CHL}_{PS_{i}}EI\_{CHL}_{CB}NOEI$) and Scheme 2 act as basic models, and we will compare the performance of other models with that of the basic models.

### 3.4. Detailed Designs of MQTT E2E Mechanisms

The four models in the previous section have a similar structure,, but with different combinations of the security function supports and of the key selections on the ${CHL}_{PS_{i}}$ channel and on the ${CHL}_{CB}$ channels; we, therefore, will not describe the details of every schemes; instead, we only detail two of them, and then list the differences for the others.

All four models share the same protocol flow in Figure 3.

**Publisher–broker connection block.** The first block, consisting of Steps 1~3, is referred to as the publisher connection block. In Step 1, a publisher randomly chooses a temporary key pair (public key, private key), sends its CONNECT request including its certificate, the temporary public key as a random challenge, and its signature on the temporary public key and on the current timestamp. The broker checks the certificate and the signature in Step 2. The broker returns its certificate and its signature on Step 3, and the publisher validates the message in Step 4. The publisher’s signature denoted as P.Sig = ${Sig}_{{PrK}_{P}}\left({TPK1}_{P},T_{P}\right)$. Likewise, the broker’s Connack response consists of its certificate, its temporary public key ${TPK1}_{B}$, its timestamp $T_{B}$, and its signature B.Sig = ${Sig}_{{PrK}_{B}}\left({TPK1}_{B},T_{B}\right)$. After validating each other’s certificate, timestamp, and signatures, they share the Diffie–Hellman publisher–broker-channel key ${SK}_{PB}=g^{{TPrK1}_{P}*{TPrK1}_{B}}mod\;p$. This channel key ${SK}_{PB}$ would be used in authentication, in integrity check, or both in the four models.

**Subscriber–broker connection E2E building block**. The second block, consisting of Steps 5~15 is referred to as the subscriber–broker connection E2E building block. In this block, a subscriber initiates its connection (and its E2E connection request) in Step 5 with its broker which processes the request and forwards the E2E request to the publisher (Step 7); Upon receiving the E2E request in Step 7, the publisher publishes its response in Step 13, and the broker processes the message and sends the Connack response to the subscriber in Step 14. If both the responses from the publisher and the broker confirm the connection request from the subscriber, the subscriber would establish one key ${SK}_{BS_{i}}$ with its broker and another key ${KE2E}_{PS_{i}}$ with its publisher. The differences of the four schemes (Scheme 1~4) in this block exist in the key establishments; In Scheme 1, ${SK}_{BS_{i}}$ is not set and ${KE2E}_{PS_{i}}$ is set as ${SK}_{SP}=g^{{TPrK2}_{S}*{TPrK2}_{P}}modp$. In Scheme 2, ${SK}_{BS_{i}}$ is set as ${SK}_{SB}=g^{{TPrK2}_{B}*{TPrK1}_{S}}modp$ and ${KE2E}_{PS_{i}}$ is set as ${SK}_{SP}=g^{{TPrK2}_{S}*{TPrK2}_{P}}modp$. In Scheme 3, ${SK}_{BS_{i}}$ is set as ${SK}_{SB}={GK}_{B}$ and ${KE2E}_{PS_{i}}$ is set as ${KE2E}_{PS_{i}}={GK}_{P}$. In Scheme 4, ${SK}_{BS_{i}}$ is set as ${SK}_{SB}={GK}_{B}$ and ${KE2E}_{PS_{i}}$ is set as ${KE2E}_{PS_{i}}={GK}_{P}$.

**Publisher–Subscriber E2E message block**. This block represents the E2E messaging phase after the E2E channel is built. In Scheme 1, both the channel ${CHL}_{PB}$ and the channel ${CHL}_{BS_{i}}$ provide no privacy and no integrity protection while the channel ${CHL}_{PS_{i}}$ is encrypted and integrity protected using ${SK}_{SP}=g^{{TPrK2}_{S}*{TPrK2}_{P}}modp$. In Scheme 2, the channel ${CHL}_{PB}$ is encrypted and integrity-protected using ${SK}_{PB}$, the channel ${CHL}_{BS_{i}}$ is encrypted and integrity-protected using ${SK}_{BS}=g^{{TPrK2}_{B}*{TPrK1}_{S}}=g^{{TPrK2}_{B}*{TPrK1}_{S}}$, and the channel ${CHL}_{PS_{i}}$ is encrypted and integrity protected using ${KE2E}_{PS_{i}}=g^{{TPrK2}_{S}*{TPrK2}_{P}}modp$. In Scheme 3, the channel ${CHL}_{PB}$ is encrypted and integrity-protected using ${SK}_{PB}$, the channel ${CHL}_{BS_{i}}$ is encrypted and integrity protected using ${SK}_{BS_{i}}={GK}_{B}$, and the channel ${CHL}_{PS_{i}}$ is encrypted and integrity protected using ${KE2E}_{PS_{i}}={GK}_{P}$. In Scheme 4, the channel ${CHL}_{PB}$ is integrity-protected using ${SK}_{PB}$, the channel ${CHL}_{BS_{i}}$ is integrity-protected using ${SK}_{BS_{i}}={GK}_{B}$, and the channel ${CHL}_{PS_{i}}$ is encrypted and integrity protected using ${KE2E}_{PS_{i}}={GK}_{P}$.

#### 3.4.1. The Details of Scheme 1

In this section, we detail every step of Scheme 1.

**Block 1**.

Step 1. The publisher selects a random number ${TPrK1}_{P}$, computes ${TPK1}_{P}=g^{\;{TPrK1}_{P}}modp$, and sends its CONNECT request, which contains ${TPK1}_{P}$ (as a challenge), timestamp $T_{P}$, its signature on ${TPK1}_{P}$ and $T_{P}$, and its long-term certificate.
  Step 2. Upon receiving the connection request, broker B stores the data in its database.
  Step 3. B selects a random number ${TPrK1}_{B}$, computes ${TPK1}_{B}=g^{\;{TPrK1}_{B}}modp$, and sends back its connect response (Connack), which contains ${TPK1}_{B}$ (as a challenge), timestamp $T_{B}$, its signature on ${TPK1}_{P}$, ${TPK1}_{B},\;T_{P}$, $T_{B}$ and its long-term certificate. B also computes the key ${SK}_{PB}=g^{{TPrK1}_{B}*{TPrK1}_{P}}modp$. This session key ${SK}_{PB}$ would be used as the encryption key and the hash key in the publisher–broker channel.
  Step 4. P receives and verifies the response, and computes the key ${SK}_{PB}$.

**Block 2.**

Step 5. The subscriber selects two random numbers ${TPrK1}_{S}$ and ${TPrK2}_{S}$, computes ${TPK1}_{S}=g^{\;{TPrK1}_{S}}modp$ and ${TPK2}_{S}=g^{\;{TPrK2}_{S}}modp$, and sends its connection request (Connect) to its broker. This connect packet contains ${TPK1}_{S}$, ${TPK2}_{S}$, timestamp $T_{S}$, its signature on the previous data, its certificate, and *its intention for building an E2E channel.*
  Step 6. B receives the request, verifies the signature, and stores ${TPK1}_{S}$, and the certificate.
  Step 7. B forwards the rest of the connect request to the publisher.
  Step 8. Upon receiving the E2E connect request, P verifies the signatures and stores ${TPK2}_{S}$ and the certificate.
  Step 9. Broker B sends its “Auth Continue” back to the subscriber. This “Auth Continue” aims to notify the subscriber that the previous connection request is still processing and please keep waiting.
  Step 10~12. Broker B and the subscriber S keep on the notification and waiting state.
  Step 13. P randomly selects ${TPrK2}_{P}$, computes ${TPK2}_{P}=g^{\;{TPrK2}_{P}}modp$, and calculates the E2E key ${KE2E}_{PS}=g^{{TPrK2}_{P}*{TPrK2}_{S}}modp$. It sends back its response, which contains its timestamp, ${TPK2}_{P}$, its signature, and its certificate.
  Step 14. B checks the content and computes ${SK}_{BS}=g^{{TPrK2}_{B}*{TPrK1}_{S}}modp$. It then forwards P’s response back to the subscriber.
  Step 15. S receives the response, verifies the signature, and computes two keys: ${KE2E}_{PS}=g^{{TPrK2}_{P}*{TPrK2}_{S}}modp$ and ${SK}_{BS}=g^{{TPrK2}_{B}*{TPrK1}_{S}}modp$.

**Block 3**

In Scheme 1, only the E2E channel ${CHL}_{PS_{i}}$ enables encryption and integrity, but the channel ${CHL}_{PB}$ and the channel ${CHL}_{BS_{i}}$ do not enable encryption/integrity. So, when the publisher P publisher a message Mtxt, it encrypts Mtxt as Ctxt = $E_{{KE2E}_{PS}}$(Mtxt), and generates the corresponding ${Hval=HMAC}_{{KE2E}_{PS}}$(Ctxt). It specifies the topic, Ctxt, Hval, etc., as an MQTT message. The broker checks the topic and forwards it to the subscriber, who uses the key ${KE2E}_{PS}$ to decrypt and verify the content.
  Scheme 1 provides the authentication, encryption, and integrity check on the E2E channel ${CHL}_{PS_{i}}$, and provides mutual authentication between clients (publisher/subscriber) and the broker. But it does not provide message authentication and an integrity check for the message between clients and the broker.

#### 3.4.2. The Details of Scheme 2

The steps 1~15 of Scheme 2 are the same as those of Scheme 1, but they differ in the steps of Block 3. When the publisher P publishes a message Mtxt, it encrypts Mtxt as Ctxt = $E_{{KE2E}_{PS}}$(Mtxt), and generates the corresponding ${Hval=HMAC}_{{KE2E}_{PS}}$(Ctxt). This process is for securing the content on the E2E channel ${CHL}_{PS_{i}}$. P further encrypts Ctxt as ${\mathrm{Ctxt}}^{\prime }=E_{{SK}_{PB}}$(Ctxt), appends it with the corresponding ${\mathrm{HAMC}}^{\prime }$, and finally specifies the topic, ${\mathrm{Ctxt}}^{\prime }$, and ${\mathrm{HAMC}}^{\prime }$ in an MQTT message to the broker.

The broker decrypts ${Ctxt}^{\prime }$ to get Ctxt, and verifies ${HAMC}^{\prime }$, using ${SK}_{PB}$. If they succeed, B further re-encrypts Ctxt as $Ctxt”=E_{{SK}_{PB}}$(Ctxt), appends a new HMAC”, and then forwards the MQTT message to the subscriber.

The subscriber S first decrypts and verifies the message, using the key ${SK}_{BS}=g^{{TPrK2}_{B}*{TPrK1}_{S}}modp$. It gets the content Ctxt and Hval, and then further decrypts it and verifies it, using the key ${KE2E}_{PS}=g^{{TPrK2}_{P}*{TPrK2}_{S}}modp$.

Scheme 2 provides mutual authentication, message authentication, and message integrity check on both the E2E channel and the client-broker channels. The encryption on the client–broker channels is based on the individual session key.

#### 3.4.3. The Details of Scheme 3

Steps 1~15 of Scheme 3 are similar to those of Scheme 1, but they differ in the selections of the channel keys and the steps of Block 3. The E2E channel ${CHL}_{PS_{i}}$ uses ${KE2E}_{PS_{i}}={GK}_{P}$, where the ${GK}_{P}$ key is set as the group key distributed by P; the channel ${CHL}_{BS_{i}}$ uses the key ${SK}_{BS_{i}}={GK}_{B}$, where ${GK}_{B}$ is the group key distributed by *B.*

When the publisher P publishes a message Mtxt, it encrypts Mtxt as Ctxt = $E_{{GK}_{P}}$(Mtxt), and generates the corresponding ${Hval=HMAC}_{{GK}_{P}}$(Ctxt). This process is for securing the content on the E2E channel ${CHL}_{PS_{i}}$. P further encrypts Ctxt as ${\mathrm{Ctxt}}^{\prime }=E_{{SK}_{PB}}$(Ctxt), appends it with the corresponding ${\mathrm{HAMC}}^{\prime }$, and finally specifies the topic, ${\mathrm{Ctxt}}^{\prime }$, and ${\mathrm{HAMC}}^{\prime }$ in an MQTT message to the broker.

The broker decrypts ${Ctxt}^{\prime }$ and verifies ${HAMC}^{\prime }$, using ${SK}_{PB}$. If they succeed, B further re-encrypts Ctxt as $Ctxt”=E_{{GK}_{B}}$(Ctxt), appends a new HMAC”, and then forwards the MQTT message to the subscriber.

The subscriber S first decrypts and verifies the message, using the key ${GK}_{B}$. It gets the content Ctxt and Hval, and then further decrypts it and verifies it, using the key ${GK}_{P}$.

Scheme 3 provides mutual authentication, message authentication, and message integrity check on both the E2E channel and the client–broker channels. The operations on the client–broker channels are based on the group key. Note that both the E2E channel and the channel ${CHL}_{BS_{i}}$ use the corresponding group keys: this can reduce some overhead, as the publisher can do the encryption/integrity protection once for all subscribers of the same message; it applies to the broker’s operations too.

#### 3.4.4. The Details of Scheme 4

Steps 1~15 of Scheme 4 are similar to those of Scheme 1, but they differ in the selections of the channel keys and the steps of Block 3. The E2E channel ${CHL}_{PS_{i}}$ uses ${KE2E}_{PS_{i}}={GK}_{P}$, where the ${GK}_{P}$ key is set as the group key chosen and distributed by P; the channel ${CHL}_{BS_{i}}$ uses the key ${SK}_{BS_{i}}={GK}_{B}$, where ${GK}_{B}$ is the group key chosen and distributed by *B.*

When the publisher P publishes a message Mtxt, it encrypts Mtxt as Ctxt = $E_{{GK}_{P}}$(Mtxt), and generates the corresponding ${Hval=HMAC}_{{GK}_{P}}$(Ctxt). This process is for securing the content on the E2E channel ${CHL}_{PS_{i}}$. Please note that P *does not* further encrypt Ctxt but only appends it with the corresponding ${\mathrm{HAMC}}^{\prime }$, and finally specifies the topic, Ctxt, and ${\mathrm{HAMC}}^{\prime }$ in an MQTT message to the broker.

The broker verifies ${HAMC}^{\prime }$, using ${SK}_{PB}$. If they succeed, B *does not* re-encrypt Ctxt but appends a new HMAC”, and then forwards the MQTT message to the subscriber.

The subscriber S first verifies the message, using the key ${GK}_{B}$. It gets the content Ctxt and Hval, and then further decrypts it and verifies it, using the key ${GK}_{P}$.

Scheme 4 provides mutual authentication, message authentication, and message integrity check on both the E2E channel; but it provides only message authentication and integrity checks on the client–broker channels. The operations on the client–broker channels are based on the group key. Note that both the E2E channel and the channel ${CHL}_{BS_{i}}$ use the corresponding group keys: this can reduce some overhead, as the publisher can perform the integrity protection once for all subscribers of the same message; it applies to the broker’s operations too. Please note that Scheme 4, compared to Scheme 3, further eliminates the double-encryption issue on the client–broker channels.

## 4. Performance Analysis, Implementations, and Evaluations

To evaluate the performance of the schemes, we first analyze the performance in Section 4.1, and then introduce our implementations and simulation evaluations in Section 4.2.

### 4.1. The Performance Analysis

Table 2 sorts out the performances in terms of the security supports, the computational performance, and the latency performance. Assume there are *n* subscribers for one MQTT message from a publisher. Let $T_{E}$ denote the computational cost for one MQTT message encryption; $T_{H}$ denotes the computational cost for one MQTT message HAMC calculation.

From Table 2, we can see that Scheme 2 demands the most expensive computational cost, due to the usage of individual keys. Scheme 4, compared to Scheme 3, slightly improves the computational overhead while keeping the necessary security supports. Even though Scheme 1 is the most efficient one in the above four schemes, Scheme 1 does not provide message authentication on the client-broker channels; therefore, Scheme 4 is the one that can achieve efficiency performance while providing the desirable security.

### 4.2. The Implementations, Simulations, and Evaluations

We implement the four schemes, using Mosquitto [37] APIs, OpenSSL [38], json-c library [39] and VMware [40]. Mosquitto is a popular open-source MQTT package, and the newest versions support MQTT 5.0. Mosquitto’s plugins facilitate developers to implement customized programs. The clients from the Paho [41] package have been adopted.

**Figure 4:**
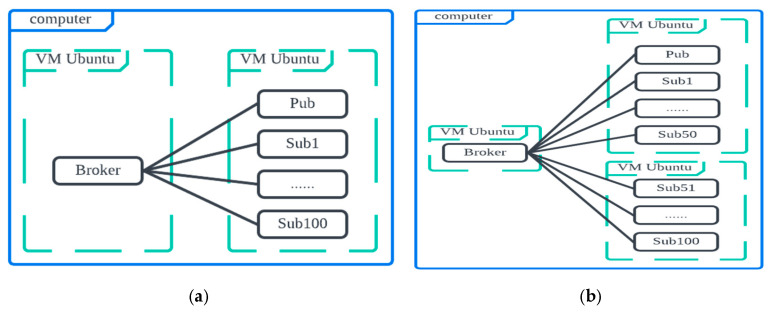
Simulation scenarios (**a**) 1 NB with 2 VMs, (**b**) 1 NB with 3 VMs.

We first design and deploy two scenarios in Figure 4. In Figure 4a (Scenario 1), there is one NoteBook (NB) with two VMs: VM1 is for the broker, and VM2 is for one publisher with 10/20/50/100/200 subscribers. In Figure 4b (Scenario 2), there is one NB with three VMs: VM1 is for the broker, and VM2 is for one publisher with 50 subscribers, VM 3 is for 50 subscribers. The hardware and software used are summarized in Table 3.

The experiments are conducted as follows. First, the broker starts up, the publisher connects, and then the subscriber connects. Second, the publisher publishes 1000 MQTT messages, and all the subscribers receive the messages. Here, we are concerned about two metrics: one is the connection latencies, and the others are the latencies between a MQTT message travels from the publisher to a subscriber.

**Connection latency**

Regarding the connection latencies, we design two scenarios: one is only five subscribers (Table 4), and the other is for a varying number of subscribers.

**Figure 5:**
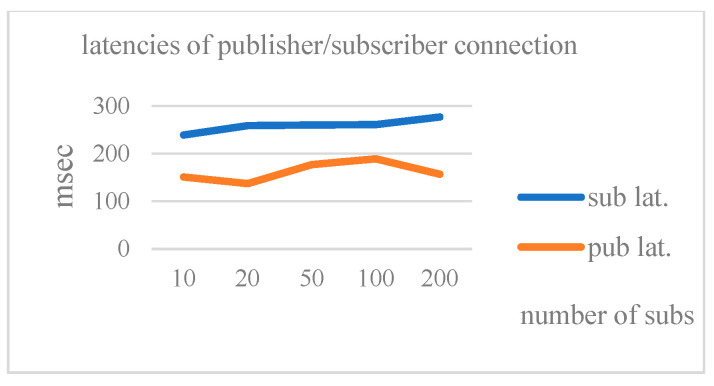
The latencies of pub/sub connections with varying number of subscribers.

Table 5 shows the connection latencies for a varying number of subscribers. Figure 5 shows the connection latencies for a varying number of subscribers. From the figure, we can see that the proposed E2E connection is quite stable, and the latency for subscribers is slightly larger than that for the publisher, as it takes more steps for the subscriber. We also notice that, even when there are 200 subscribers, the subscribers’ connection latency is still quite small (less than 300 ms). It confirms that the proposed E2E connection is quite efficient.

**Publishing–receiving latency**

**Figure 6:**
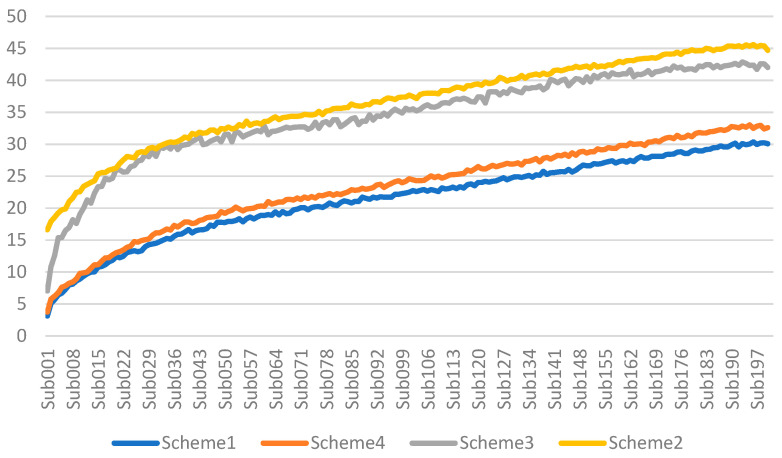
The latencies of the four schemes for different subscribers in Scenario 1.

The publishing–receiving latencies in Scenario 1 are summarized in Table 6. Figure 6 shows the latencies of the four schemes for different subscribers in Scenario 1. Here, we can see that, as the number of subscribers increases, the latencies increase in the four schemes. Comparing the latencies of the four schemes, Scheme 1 < Scheme 4 < Scheme 3 < Scheme 2, which confirms our analysis that more overhead on the client–broker channel would have an impact on the latency performance. Obviously, Scheme 2 demands the most cost (shown in Table 2) and demands the longest latency (shown in Figure 6).

Next, we focus on comparing Scheme 1, Scheme 3, and Scheme 4 for Scenario 2. Here, the results (Table 7) again confirm that the latency order is Scheme 1 < Scheme 3 < Scheme 3 < Scheme 2. In these scenarios, we find that the average latency of Scheme 4 is the smallest, and both Scheme 3 and 4 can improve the latency performance while preserving the desirable security. That is, both the group-key-based approach can improve the efficiency and the client–broker channel integrity-only approach can further reduce the average latency.

**Figure 7:**
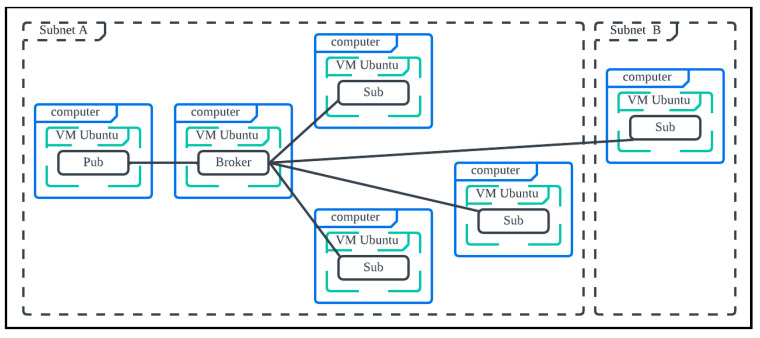
Simulation scenario: six PCs each hosting one VM (total six VMs).

Next, we focus on Scenario 3 (Figure 7), where subscribers are deployed in two different sub-nets (domains) to evaluate the inter-networking impact on the latencies.

The results (Table 8) indicate that both Scheme 3 and Scheme 4 could improve the latency performance, compared to Scheme 2 (the conventional MQTT E2E model); that is, the group-key-based approach apparently reduces the latency. In both the 100/200-subscriber simulations, the latency of Scheme 3 > the latency of Scheme 4. This indicates that the client–broker channel integrity-only approach further improves the latency.

**Figure 8:**
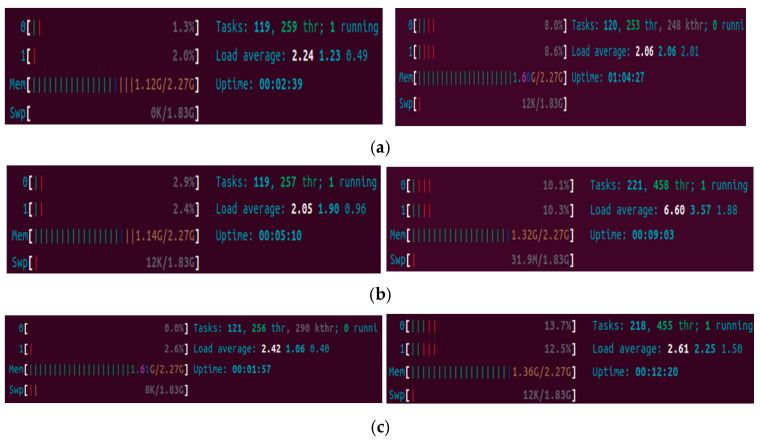
(**a**) The CPU/memory utilization of the broker VM1 before/after subscriber subscribers; (**b**) the CPU/memory utilization of the publisher-100subscriber VM2 before/after subscriber active subscribers; (**c**) the CPU/memory utilization of the100subscriber VM3 before/after subscriber/active subscribers.

Figure 8 shows the memory/CPU utilization in broker VM, the publisher-100subscriber VM2, and the 100subscriber VM3. We can see that the CPU/memory utilization for all three VMs increases after the subscribers become active: it means that the broker, the publisher, and the subscribers need to process more computations and communications.

Summarizing the above analysis and the simulations, we confirm that the proposed Scheme 4 (${CHL}_{PS_{i}}EIG\_{CHL}_{BS}IG$) can significantly improve the latency while preserving the desirable security.

### 4.3. Comparison

Here, we make a comparison among some studies which are related in a broader sense; some of them are MQTT related and some are just general pub/sub systems. Table 9 briefly summarizes the qualitative features of these schemes.

Here, we have some observations from the table. Regarding the MQTT broker-based E2E models, our work confirms that both the group key-based approach and the client–broker channel integrity-only approach can improve the efficiency while preserving the desirable security properties. When we extend the scope to include brokerless MQTT and SDN communication support, they both have the potential to improve the efficiency, too; these approaches can complement each other and combining the ideas from these approaches might further improve the overall performance; however, further investigations are required.

## 5. Security Analysis and Proof

This section will examine the security of the proposed scheme. Here, we will focus on the security analysis of Scheme 4 in Section 5.1 and the security proof in Section 5.2. Here, we focus on Scheme 4 as it satisfies the security goals and greatly improves the latency performance (as shown in Section 4).

### 5.1. Security Analysis

Here, we analyze each security goal of Scheme 4.

**The mutual authentication of the publisher–broker channel ${CHL}_{PB}$**. In the channel building ${CHL}_{PB}$ phase (Block 1), the publisher and the broker exchange their certificates, their ephemeral public keys $({TPK1}_{P}$,${TPK1}_{S})$, timestamps, and their digital signatures on the transmitted data, which enable them to authenticate the participating entities. During the authentication, they establish their Diffie–Hellman key ${SK}_{PB}=g^{{TPrK1}_{P}*{TPrK1}_{B}}\mathrm{modp}$.
**The privacy and integrity of the MQTT payload in the ${CHL}_{PB}$**
  **channel.** After the channel building, the MQTT payload would be encrypted using the Diffie–Hellman key ${SK}_{PB}$ in Schemes 2,3, which ensures the privacy of the MQTT payload in the ${CHL}_{PB}$ channel in Schemess 2,3. The MQTT payload would be integrity protected in Schemes 2,3,4.
  **The mutual authentication of the broker–subscriber channel ${CHL}_{BS}$**. In the channel building ${CHL}_{BS}$ phase (Block 2), the broker and the subscriber exchange their certificates, their ephemeral public keys $({TPK2}_{B}$,${TPK1}_{S})$, timestamps, and their digital signatures on the transmitted data, which enable them to authenticate the participating entities. During the authentication, they establish their Diffie-Hellman key ${SK}_{SB}=g^{{TPrK2}_{BR}*{TPrK1}_{S}}modp$, and the broker’s group key ${GK}_{B}$ is securely distributed in Schemes 3,4.
**The privacy and integrity of the MQTT payload in the ${CHL}_{BS}$**
  **channel.** After the channel building, the MQTT payload would be encrypted using the Diffie–Hellman key ${SK}_{PB}$ in Scheme 2 and using the group key ${GK}_{B}$ in Scheme 3, which ensures the privacy of the MQTT payload in the ${CHL}_{BS}$ in Schemes 2,3. The payload would be integrity protected in Schemes 2,3,4.
  **The mutual authentication of the publisher and the subscriber in an E2E channel**. In the E2E channel building phase, the publisher and the subscriber exchange their certificates, their ephemeral public keys $({TPK2}_{P}$,${TPK2}_{S})$, timestamps, and their digital signatures on the transmitted data, which enable them to authenticate the participating entities. After that, they can establish their E2E keys (${KE2E}_{PS_{i}}=g^{{TPrK2}_{S}*{TPrK2}_{P}}modp$ in Schemes 1,2,3,4, and ${GK}_{P}$ securely distributed and protected using the session key ${KE2E}_{PS_{i}}$ in Schemes 3,4).
**The privacy and integrity of the MQTT payload in the ${CHL}_{PS}$**
  **channel.** After the channel building, the MQTT payload in the ${CHL}_{PS}$ **channel** would be encrypted using the Diffie–Hellman key ${SK}_{PS}$ in Schemes 1,2 and using the group key ${GK}_{B}$ in Schemes 3,4, which ensures the privacy of the MQTT payload in the ${CHL}_{PS}$ in Schemes 1,2,3,4.

Summarizing all the above security analysis, we can note that Scheme 4 achieves the security functions: (1) mutual authentication in the ${CHL}_{PS},{CHL}_{PB},{CHL}_{BS}$ channels; (2) authenticity and integrity of the MQTT messages in the ${CHL}_{PB},{CHL}_{BS}$ channels; (3) privacy of the MQTT payload in the ${CHL}_{PS}$ channel.

### 5.2. Security Proofs

We specify the protocol using the HLPSL language, prove the security goals using the AVISPA, and evaluate the security performance.

**Security goals.** The security goals of the proposed scheme include: (1) the mutual authentication between a client (publisher or subscriber) and the broker, (2) and the privacy of the shared keys of the channels (${CHL}_{PB},\;{CHL}_{BS}$), (3) the mutual authentication between a publisher and a subscriber and the privacy of the E2E key.

We formally specify the protocol and its goals using the HLPSL language. The SPAN protocol simulation validates its correctness. We use both the OMFC verifier and the ATSE verifier to prove the security goals. In HLPSL, each entity is modeled as a role which interacts with other roles.

**The HLPSL modeling of MQTT E2E scheme**

**Figure 9:**
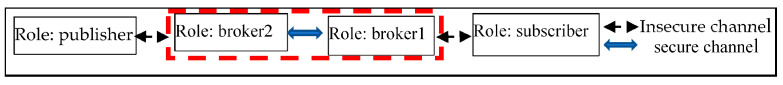
The 2-broker-HLPSL model.

In the conventional client–server architecture, both server and client can be viewed as independent finite-state machines which interact with their communicating party through messages, which is easy and intuitive to model each entity as an independent role using HLPSL. However, it is a challenge to model a single role for a broker of MQTT, because MQTT is based on the publisher–broker–subscriber architecture, where the broker independently authenticates each client, but it also handles the associations of all clients which involve the same topic. To solve this challenge, we proposed the two-broker-HLPSL model where a broker is separated into two roles, but the two roles (broker1 and broker2) securely share their information through symmetric-key-based encryptions (Figure 9). In this setting, each broker can independently handle the interactions with its client, and the two brokers can share the topic-bounded messages. There are a total of four roles—subscriber, broker1, broker2, publisher—in our MQTT HLPSL modeling. We note that, due to the limited support of exponentiation in the verifiers, we pass symmetric keys as inputs to assign the calculated CDHP keys in the specifications.

**The SPAN-OMFC simulation/verification**

**Figure 10:**
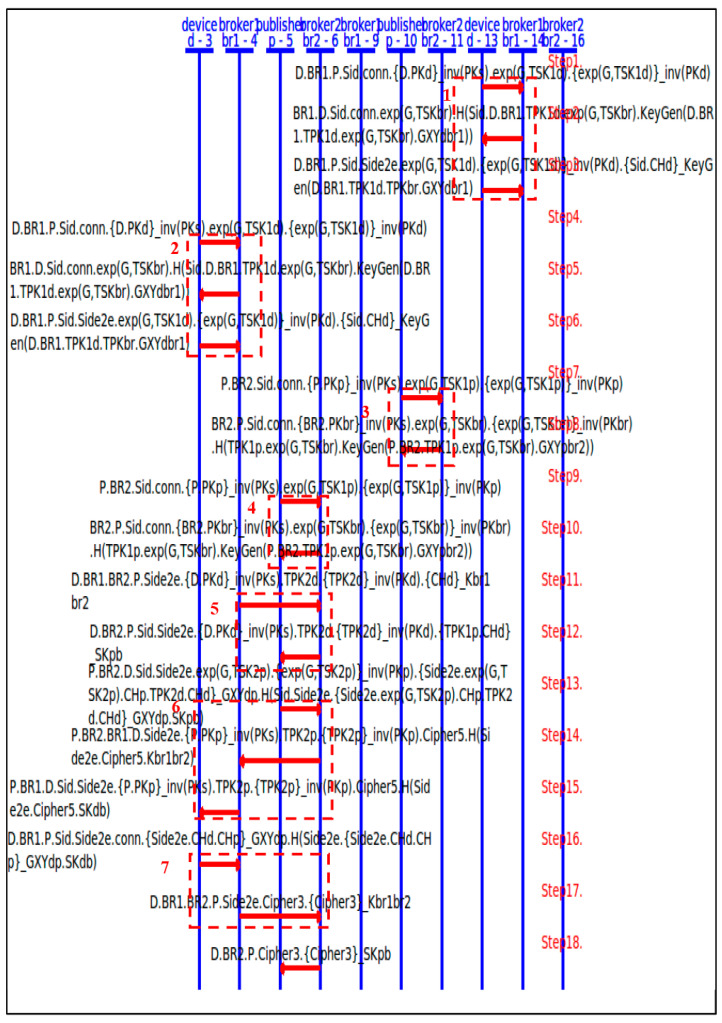
The SPAN simulation of our MQTT-E2E HLPSL specification.

Figure 10 shows the message sequences of the SPAN protocol simulation of our MQTT-E2E HLPSL specification.

The first two dashline blocks (Blocks 1, 2) show two pairs of device-broker1 performing the authentications, in which a device refers to a client. Blocks 3 and 4 show two pairs of publisher-broker2 performing the authentications. Block 5 shows broker1 and broker 2 forwarding the E2E request to the publisher, which is followed by block 6 in which the publisher sends back its E2E response with the temporary DH public key ${TPK2}_{p}$ to the subscriber. Block 7 shows the subscriber sending the confirmation back to the publisher. The SPAN simulation confirms that the roles successfully execute the specified protocol flow.

**Figure 11:**
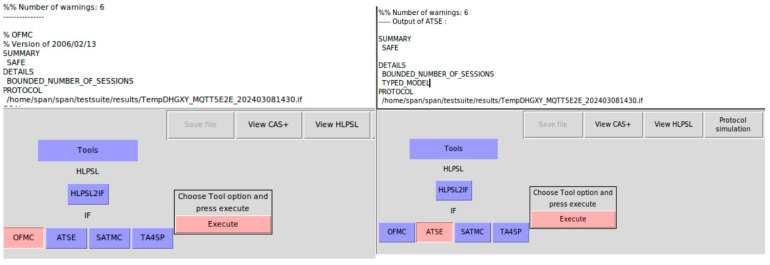
Both OFMC and ATSE confirm SAFE of our HLPSL specification.

The security goals of the specification include the mutual authentication for device–broker1 and for publisher–broker2, the publisher–subscriber authentication, and the privacy of the session keys for device–broker1/publisher–broker2/the end2end session key. We use both the OMFC verifier and the ATSE verifier to validate the security goals. Figure 11 shows both the OMFC verifier and the ATSE verifier verification results—SAFE.

## 6. Conclusions

In this paper, we have discussed the performance weaknesses of the conventional MQTT broker-based E2E model—the double-encryption issue on the client–broker channel and the heavy broker-decrypt-re-encrypt burden issue. These two issues downgrade the MQTT efficiency advantage. To tackle the challenges and to improve the efficiency of the conventional MQTT broker-based E2E model, we propose two approaches- the group-key-based approach and the client-broker-channel-integrity-only approach. To verify the efficiency performance, we have implemented the schemes and experimented with them in three scenarios. The results confirm that the two approaches could improve the efficiency while preserving the desirable security properties. The security analysis ensures Scheme 4 meets the security requirements, and the formal AVISPA toolsets formally confirm the security goals of the MQTT-E2E HLPSL specification. Some interesting future studies include the integration of the approaches we propose here and ideas like brokerless MQTT or SDN to improve the overall performance further.

## Figures and Tables

**Table 1:** The Notation.

P: Publisher; S: Subscriber; B: Broker. $S_{i}$: *it*h subscriber	${PK}_{P}/{PrK}_{P}$: long-term public/private key of *P*. Similar notations apply for *S* and *B*.
*E*()/*D*(): symmetric key encryption/decryption	${TPK1}_{P}/{TPrK1}_{P}$, ${TPK2}_{P}/{TPrK2}_{P}$: temporary public/private key of *P*. Similar notations apply for *S* and *B*. For example, ${TPK1}_{B}=g^{{TPrK1}_{B}}modp$ is a temporary private key for *B*.
$T_{P}$: Timestamp of P; *HMAC*(): message authentication code	${GK}_{P}:\;the\;group\;key\;distributed\;by\;P;\;{GK}_{B}$ *: the group key distributed by B;*
${SK}_{PB}$/${SK}_{BS_{i}}/{SK}_{SP}$: the session key between *P* and *B*/between *S_i_* and *B*/between *S* and *P.* ${SK}_{PB}=g^{{TPrK1}_{B}*{TPrK1}_{P}}modp$. ${SK}_{SB}=g^{{TPrK2}_{B}*{TPrK1}_{S}}modp$. ${SK}_{SP}=g^{{TPrK2}_{S}*{TPrK2}_{P}}modp$.	
G: the generator for the Elliptic Curve field	${KE2E}_{PS_{i}}$: the E2E-channel key between P and $S_{i}$
${Sig}_{{PrK}_{P}}\left(\right):$ signature by *P*.	${CHL}_{PB}/{CHL}_{BS_{i}}$: the channel between P and B/between B and $S_{i}$. ${CHL}_{PS_{i}}$: the E2E channel between P and $S_{i}$.
EI: Encryption with Integrity; NOEI: No Encryption and NO Integrity; E: Encryption only; I: Integrity only.	

**Table 2:** Functional performances of the four schemes.

Schemes	Scheme 1	Scheme 2	Scheme 3	Scheme 4
P-B channel security	NoEI	EI with individual key	EI with individual key	I with individual key
B-S channel security	NoEI	EI with individual key	EI with group key	I with group key
P-S channel security	EI with individual key	EI with individual key	EI with group key	EI with group key
P’s computational complexity for 1 MQTT msg.	$n*(T_{E}+T_{H})$	$\left(n+1)*(T_{E}+T_{H}\right)$	$2T_{E}+2T_{H}$	$1T_{E}+{2T}_{H}$
B’s computational complexity for 1 MQTT msg.	$1T_{E}+1T_{H}$	$\left(n+1)*(T_{E}+T_{H}\right)$	$2T_{E}+2T_{H}$	${2T}_{H}$
S’s computational complexity for 1 MQTT msg.	$1T_{E}+1T_{H}$	$2T_{E}+{2T}_{H}$	$2T_{E}+2T_{H}$	$1T_{E}+{2T}_{H}$

P: Publisher; B: Broker; S: Subscriber; NoEI: No Encryption and Integrity; E: Encryption; I: Integrity; Msg: message.

**Table 3:** The hardware and the software used in the simulations.

host machine	CPU: Intel^®^ Core™ i7-8700 CPU @ 3.20 GHz, 6 cores, 12 threads Motherboard: Dell 0PXWHK; RAM: 64 GB; GPU: NVIDIA GeForce GTX 1070
VM	2 vCPUs & 4 GB RAM
Software	Ubuntu 22.04.5 LTS, VMware® Workstation 17 Pro 17.6.3, Paho-mqtt 1.6.1, Mosquitto 2.0.15, Openssl 3.0.2, json-c 0.16.99

**Table 4:** Summary of 50 connection latencies of publishers and five subscribers (ms).

	Pub	Sub 1	Sub 2	Sub 3	Sub 4	Sub 5	
**AVERAGE**	135	163	142	155	150	159	AVG(Sub1~5) = 153.8
**MAX**	191	282	149	186	186	193	MAX(Sub1~5) = 282
**MIN**	39	57	52	74	50	47	MIN(Sub1~5) = 47
**MAX(w)**		4	1	1	1	1	

w = Wait count, w = 1(+25 ms), w = 2(+50 ms); Pub: publisher, Sub: subscriber.

**Table 5:** The latencies of pub/sub connections with a varying number of subscribers.

Number of Subs	10	20	50	100	200
Latencies of pub/sub	151/239	137/259	177/260	189/261	157/277

**Table 6:** The publishing–receiving latencies in Scenario 1.

Publish–Receive Delay	Average	Max	Min	Median	StdDev	Variance
Scheme 1	21.44	115.50	0.67	21.82	9.14	83.61
Scheme 4	23.30	169.94	0.74	23.40	10.30	106.03
Scheme 3	34.42	154.49	0.88	36.82	14.35	205.88
Scheme 2	36.51	153.63	7.46	36.58	12.54	157.33

**Table 7:** Publish–receive latencies in Scenario 2.

	Scheme	Average	Max	Min
sub 100	Scheme 1	9.856	84.174	0.627
	Scheme 4	10.399	96.933	0.809
	Scheme 3	12.587	88.264	0.861
	Scheme 2	17.239	111.122	3.669
sub 200	Scheme 1	15.939	100.076	0.663
	Scheme 4	17.299	204.405	0.727
	Scheme 3	21.728	100.141	0.929
	Scheme 2	30.418	109.806	7.597

**Table 8:** Publish–receive latencies in Scenario 3.

	Scheme	Average	Max	Min
sub 100	Scheme1	15.079	440.975	1.671
	Scheme4	15.013	433.278	2.129
	Scheme3	16.763	242.792	2.395
	Scheme2	26.798	274.494	6.899
sub 200	Scheme1	24.839	278.347	1.705
	Scheme4	26.407	279.038	2.111
	Scheme3	28.783	406.476	2.16
	Scheme2	57.255	360.384	13.643

**Table 9:** Qualitative comparison among pub/sub systems and MQTT mechanisms.

Scheme/Approach	E2E Key Establishment	Main Challenges/Limitations
Mektoubi et al. [7]	Topic certificate’s public/private key to encrypt/decrypt topic messages	Very huge overhead from the clients; the challenges of private key distribution
MQTLS [4]	OpenSSL-based extensions are adopted to support D-H E2E key negotiated between publisher/subscriber.	MQTT 5.0 compatibility is not considered. Broker-based group key is not considered. Double-encryption issue is not considered.
SEEMQTT [5]	A publisher delegates the message decryption key to a pool of key stores. Subscribers contact the key pools; they get verified and get the decryption key, via the secret sharing mechanism.	A lot of extra overhead of verification and key derivation might compromise the efficiency of MQTT.
Eugster et al. [9]	Discussion the decoupling properties of the publish/subscribe systems.	No specific schemes or approaches are proposed or suggested.
MQTT-I [14]	Combining digest hashing, multiple-topic Merkle hash tree, and round-based signature/verification to amortize the overhead and to ensure the message flow integrity of MQTT E2E channel.	E2E privacy against a curious broker is not considered. The impact of the increasing number of clients on MQTT systems is not considered.
Park-Nam [16]	The E2E key (topic key) is derived from the secret D-H key calculation of the topic public key and the publisher’s secret key (or of the topic private key and the publisher’s public key.	A publisher can only publish one topic. The group-key-based approach is not applicable to enhance efficiency, as all the session keys are tightly dependent on each client’s private key.
Ciphertext-matching like [23]	Concern the event privacy and the subscription privacy against a curious broker for a general pub/sub system. Encrypted subscription matching is applied for matching and routing.	Even though MQTT E2E also concern the message privacy against a curious broker, both the subscribed topics and the topic of a published message are explicitly sent to the broker in MQTT systems.
Brokerless approach like [24,25,26]	The brokerless approach utilizes SDN and/or/User properties to bypass brokers and reserve network bandwidth to improve latency performance.	Our approach aims to improve efficiency by adjusting the MQTT broker-based E2E models, while the broker-less approach adopts transmission reservation facilities like SDN to improve transmission latency. These two approaches are complementary; the ideas from either approach can be combined to improve efficiency further.
Attribute-based encryption [17,18,19]	Key/Ciphertext Policy-Attribute Based Encryption (KP/CP-ABE) is adopted to design the session key.	This approach has a key weakness of poor performance and scalability, especially when the number of attributes increases; this weakness should be seriously tackled, as MQTT is designed for IoT systems where the number of clients and the number of topics are scalable.
Our work	Focus on the double-encryption issue and the broker-decrypt-re-encrypt issue on the MQTT broker-based E2E models. Both the group-key-based approach and the client–broker channel, integrity-only approach can improve efficiency while preserving the desirable security properties.	Brokerless MQTT approach is not considered. SDN extension is not considered. No specific group key management is proposed.

## Data Availability

The raw data supporting the conclusions of this article will be made available by the authors on request.
